# Overexpression of Mineralocorticoid Receptors Partially Prevents Chronic Stress-Induced Reductions in Hippocampal Memory and Structural Plasticity

**DOI:** 10.1371/journal.pone.0142012

**Published:** 2015-11-23

**Authors:** Sofia Kanatsou, Brenna C. Fearey, Laura E. Kuil, Paul J. Lucassen, Anjanette P. Harris, Jonathan R. Seckl, Harm Krugers, Marian Joels

**Affiliations:** 1 Department of Translational Neuroscience, Brain Center Rudolf Magnus, University Medical Center Utrecht, Utrecht, The Netherlands; 2 Swammerdam Institute for Life Sciences, Center for Neuroscience, University of Amsterdam, Amsterdam, The Netherlands; 3 Endocrinology Unit, Centre for Cardiovascular Science, Queen's Medical Research Institute, the University of Edinburgh, Edinburgh, United Kingdom; Technion - Israel Institute of Technology, ISRAEL

## Abstract

Exposure to chronic stress is a risk factor for cognitive decline and psychopathology in genetically predisposed individuals. Preliminary evidence in humans suggests that mineralocorticoid receptors (MRs) may confer resilience to these stress-related changes. We specifically tested this idea using a well-controlled mouse model for chronic stress in combination with transgenic MR overexpression in the forebrain. Exposure to unpredictable stressors for 21 days in adulthood reduced learning and memory formation in a low arousing hippocampus-dependent contextual learning task, but enhanced stressful contextual fear learning. We found support for a moderating effect of MR background on chronic stress only for contextual memory formation under low arousing conditions. In an attempt to understand potentially contributing factors, we studied structural plasticity. Chronic stress altered dendritic morphology in the hippocampal CA3 area and reduced the total number of doublecortin-positive immature neurons in the infrapyramidal blade of the dentate gyrus. The latter reduction was absent in MR overexpressing mice. We therefore provide partial support for the idea that overexpression of MRs may confer resilience to the effects of chronic stress on hippocampus-dependent function and structural plasticity.

## Introduction

Human studies have shown that frequent exposure to stressful events is an important risk-factor for developing psychopathology, especially in individuals with a predisposing genetic background [1–3]. This supports the notion that gene–environment interactions affect susceptibility to stress-related diseases, with some genes enhancing susceptibility and others conferring resilience to stressful events in adulthood [4–5].

Upon exposure to stressful events, the adrenal glands secrete high levels of corticosteroid hormones (corticosterone in rodents and cortisol in humans; [4–5]. Corticosteroids enter the brain and bind to two types of intracellular receptors: mineralocorticoid (MRs) and glucocorticoid receptors (GRs). MR and GR exert slow transcriptional control over responsive genes via hormone response elements present in promoters [5]. Apart from their actions in the nucleus, MR and GR also mediate rapid, non-genomic actions via membrane-located receptors [6].

GRs are present throughout the brain and have a low affinity for corticosterone [5]. Under non-stressful conditions only part of the receptors are occupied, whereas stress-levels of corticosteroids cause a substantial increase in binding. Intracellular MRs are highly expressed in some limbic areas, notably hippocampus and amygdala, and have a much higher affinity for corticosterone, implying substantial occupancy even by basal hormone levels. Increasing MR density may thus more effectively change MR signaling than increased ligand concentrations. The affinity of membrane-located MRs appears to be similar to that of GRs [7].

MRs play a crucial role in synaptic transmission and plasticity. For instance, non-genomic MR actions facilitate glutamatergic transmission in the hippocampus and amygdala and enhance synaptic retention of AMPA receptors [8–10]. Behaviorally, MRs promote an appropriate learning/cognitive strategy under stressful conditions and are relevant for spatial learning or establishing contextual fearful memories [11–13]. In addition, MR overexpression in the forebrain reduces anxiety and enhances memory retention [14–15].

Studies in humans also support the importance of MRs for behavioral adaptation. First, splice variants of MR mRNA and *lower* levels of mRNA were found in postmortem brain regions of patients with major depressive disorder [16]. Second, in an aged population, carriers of the MR-I180V single-nucleotide polymorphism (SNP) associated with loss of function had a higher prevalence of depressive symptoms than non-carriers [17]. Third, a specific MR haplotype–related to *high* MR expression levels- was associated with a heightened dispositional optimism in women [16]. In agreement, a recent study in humans revealed a beneficial effect of MR stimulation in cognition both in depressed patients and healthy individuals [18].

Moreover, a human study using fMRI has shown that a common functional polymorphism of the MR (Iso /Val (rs5522), associated with variability in HPA axis reactivity, moderates the effect of childhood emotional neglect and amygdala reactivity [19]. In stressed individuals, a shift from hippocampal to dorsal striatal memory systems was found to be dependent on the MR activation [20]. Also, blockade of MRs prevents the stress-induced enhanced connectivity between the amygdala and the dorsal striatum, possibly explaining the behavioral shift towards habitual responses, and prevents a stress-dependent shift from trace to delay conditioning learning [21–22].

These studies have led to the hypothesis that high (functional) MR levels confer resilience to stress-related psychopathologies [23,15]. In our study, we tested this hypothesis in a mouse model. Specifically, we examined if enhanced forebrain MR expression levels confer resilience to the effects of chronic unpredictable stress regarding hippocampus-dependent behavior and hippocampal structural plasticity.

Since activation of corticosteroid receptors depends on plasma corticosterone levels [5, 24], MRs may play a different role in behavioral tasks which differ in the degree of salience. We therefore assessed the effects of chronic unpredictable stress and MR overexpression on behavior under low arousing and high arousing experimental conditions, which differ in the degree of activation of the HPA-axis.

## Materials and Methods

### Animals

All mice used in our experiments were bred in-house. In each breeding cage, two wild type C57Bl6 female mice (Harlan, The Netherlands) were housed with one MR-transgenic (MR-tg) male mouse for one week [14]. At postnatal day 23, all pups were weaned, genotyped and pups with identical genotypes but from different litters housed two per cage (either MR-Tg or their littermate controls). Mice were kept in a temperature and humidity controlled facility (21.5–22°C, with humidity between 40 and 60%) on a 12h light/dark cycle (lights on at 8:00 a.m.) with food and water available *ad libitum*. All experiments were performed in strict accordance with the Dutch regulations for animal experiments. The protocol was approved by the committee on Animal Health and Care from the University of Amsterdam, the Netherlands (Permit Number: DED 206) and in accordance with the EC Council Directive of September 2010 (2010/63/EU). All efforts were made to minimize suffering.

### Chronic Unpredictable Stress

At two months of age, mice were randomly selected for chronic unpredictable stress (CUS) or control treatment. Mice in the CUS group were exposed to a 21-days CUS paradigm that was based on earlier described approaches [25–27]. The stressors used in this CUS paradigm were applied daily (each different stressor was repeated once-weekly) in an unpredictable order between 8:30 a.m. and 12:30 p.m. in a separate room. Control mice were left undisturbed in their home cages in the regular housing room.

The following stressors were used in the CUS protocol:

Elevated plus maze (EPM): All mice were placed one-by-one in the center of a light grey colored plus formed maze (UGO BASILE S.r.l.–Italy) always facing the same open arm. After 15 minutes, the mice were removed from the maze and returned to their home cages. The maze was cleaned thoroughly with 70% EtOH between each use. All movements of the mice were recorded using a life-recording camera system that was installed above the maze (Ethovision XT 6, the Netherlands). The EPM was performed on day 1, day 11 and day 21 of the CUS regimen and only in animals that underwent CUS. We applied the EPM on day 1 to test the anxiety levels of both MR-tg and control stressed animals (at that time non-stressed, as this is the first stressor of the regimen) and on day 21 to test for habituation over time (in the stressed animals only) (S1 Fig).

Forced swim: Mice were placed for 3 minutes in a glass beaker (24 cm x 12 cm x 12 cm, Fisher Scientific, the Netherlands) filled with 1.6L water at 28°C. After these 3 minutes, the mice were placed in a cage under a red lamp for 5 minutes in order to dry before returning them to their home cages.

Rotating platform: Home cages with two mice were placed on a see-saw rocker for 1 hour with 70 oscillations per minute.

Wet sawdust: Four mice were placed in a cage filled with a layer of wet sawdust for 4 hours.

Immobilization: Mice were individually placed in a restrainer for 30 minutes.

Group housing: Four mice (two MR-tg and two control animals) were housed overnight (5:00 p.m. until 9:00 a.m.) in a single cage.

Isolation: Mice were housed overnight individually in a type 2 cage (140 mm x 365 mm x 207 mm).

### Neuroendocrine parameters

To evaluate the effectiveness of CUS, we measured bodyweight, thymus weight and adrenal weight. The bodyweights of the mice were recorded at three time-points: three hours after the stressor on day 1, three hours after the stressor on day 11 and in the morning of day 22 (one day after the CUS).

To further validate the effectiveness of CUS, a subset of mice was decapitated on day 22 between 08.30 and 11.00 h, and trunk blood was collected in EDTA-covered capillary tubes (Sarstedt, The Netherlands) to measure basal plasma corticosterone levels. These levels were measured in duplicate via a radioimmunoassay kit according to the manufacturer’s protocol (MP Biochemicals, Amsterdam, The Netherlands). At the same time-point, the adrenal glands and thymus were dissected and weighed.

### Behavior

We started all behavioral tests one day after the final stressor of the chronic unpredictable stress (PND82) and during the light phase (9:00 a.m. and 13:00 a.m.) using different cohorts of mice 1) for memory in low arousing behavioural tasks (object in-context) and 2) for memory under high-arousing conditions (contextual fear conditioning). As the elevated plus maze is one of the stressors of the CUS paradigm, we used the data of the stressed animals only (from both cohorts that underwent behavioral testing as mentioned above) to assess anxiety and locomotion upon initiation of the CUS (day1) and during the last day of the CUS (day21, S1 Fig).

#### Object in-context memory

We tested one batch of mice for place memory in a non-stressful test specific for the influence of context on object recognition [28–30]. As a context we used blue-colored plastic boxes (h x l x w: 33 cm x 54 cm x 37 cm) that contained bedding material; blocks of Lego and small bottles were used as objects. The overall test was performed over three days (illustrated in Fig 3): on day 1 the mice were habituated for 10 minutes in the context without objects. On day 2 the mice habituated first for 10 minutes in a context (A) that had no cues on the walls but had two identical objects, i.e. 2 blocks of Lego, placed in opposite corners. After this, mice were habituated for another 10 minutes in a novel context (B) that had cues on the walls in the form of stripes and contained two (new) identical objects, namely 2 small bottles, placed in opposite corners. Between habituating to both contexts, a retention interval of 1 min was used, during which the mice were kept in a familiar cage (i.e. a cage that was used to transfer the mice from the housing room to the testing room) until context B was presented. On day 3 the object in-context recognition memory was tested by placing the mice for 10 minutes in context B in which 1 of the 2 familiar objects of this context, namely the small bottle, was replaced by an object from context A, i.e. a block of Lego.

In all contexts, the mice were placed facing the same wall and opposite to the objects. Objects were cleaned thoroughly, placed in a 15 cm distance from the corners of each context and new bedding material was added on top of the old and mixed between each task, in order to saturate the olfactory cues of previous mice that had been tested. Sniffing was used as explorative behavior when the animal displayed such behavior towards an object within a distance of maximum 2 cm. Climbing on top of or watching the objects from a (close) distance was not considered as sniffing behavior.

We used the discrimination index (DI) observed on day 3 as measurement of the object in-context recognition memory. The DI is calculated as time being spent exploring the novel object compared to the total exploration time towards both objects (tnovel /(tnovel + tfamiliar)) [31].

#### Contextual fear conditioning

Contextual fear was also assessed one day after the final stressor, in another batch of mice. On day 1, the mice were placed in a chamber that had a stainless steel grid floor connected to a shock generator [13]. Mice were allowed to explore the context for three minutes after which they received a single mild foot shock (0.4 mA) for 2 seconds. After this shock, the mice stayed for an additional 30 seconds in this chamber. On day 2, the mice were placed in the same chamber and their freezing behavior was recorded for 3 minutes.

The freezing behavior of the mice was scored every 2 seconds on both days in each chamber, with ‘freezing’ being defined as ‘no body movements except those related to breathing’ [32].

### Pyramidal cell morphology

To examine the effects of stress on the morphology of hippocampal pyramidal neurons we performed Golgi-Cox staining as previously described [33–35]. Next, using yet another batch of mice killed in the morning one day after the final stressor, brains were embedded in celloidine and cut into 200 μm thick sections using a vibratome.

Using a light microscope (LSM510, Zeiss, Germany), Z-stacks (step size 1μm) from each neuron were collected in bright field mode using a light microscope (LSM510, Zeiss, Germany) with a 20x magnification. Full images of the dendritic tree of Golgi-stained neurons were reconstructed in ImagePro equipped with the Neurodraw toolbox (kindly provided by G. Ramakers, J. Van Heerikhuize and C. Pool, The Netherlands Institute of Neuroscience, Amsterdam).

For analysis, we used 5–8 pyramidal neurons per mouse present exclusively in the CA3 hippocampal area along the rostral-caudal hippocampus. We used selection criteria for each neuron as previously described [36]. Earlier studies have shown that apical rather than basal dendritic trees are very sensitive to chronic stress [37]. Therefore, we calculated the total dendritic length, the number of branch points and dendritic complexity index of the apical dendrites of every reconstructed pyramidal cell. In addition, we performed a 3D Sholl analysis using the software package NeuronStudio [33, 38].

### Neurogenesis

Neurogenesis has been implicated in cognition, fear and behavioral flexibility [39] and is regulated by stress and altered after MR modulation [40, 41].

To study the long-term effect of CUS on hippocampal neurogenesis, we performed immunostaining using three different markers in another batch of mice, killed one day after the final stressor: 1) Bromodeoxyuridine (BrdU) as an indicator for cell-survival [42–43]; 2) Ki67 to examine cell-proliferation [38, 43]; and 3) doublecortin (DCX) to identify early-stage differentiating neurons [44].

BrdU-labeling was assessed by administering a total dose of 300 mg/kg BrdU ((Sigma-Aldrich, St. Louis, Missouri, USA) 10mg/ml in 0.007 M NaOH/0.9% NaCl) intraperitoneally via three individual injections of 100 mg/kg with an interval of two hours between each injection three hours after the first stressor [38,45].

#### Perfusion

Twenty four hours after the final stressor, mice were anaesthetized and transcardially perfused with a paraformaldehyde solution (4% paraformaldehyde in 0.1M phosphate buffer pH7.4). Next, the brains were extracted and post-fixed overnight at 4°C and subsequently cryoprotected in a sucrose solution (30% sucrose in 0.1M phosphate buffer, pH7.4) on a shaking platform at 4°C for one day.

#### Immunohistochemistry

Perfused brains were sectioned into 40 μm thick sections and stored at -20°C. Sections selected for immunohistochemistry were first overnight incubated at 4°C on a rocker with the primary antibodies: rat monoclonal anti-BrdU (CloneBU1/75 (ICR1), Accurate Chemical; 1:500)) [46–47], rabbit polyclonal anti-Ki-67 (NCL-L-Ki67_MM1, Novocastra, Newcastle upon Tyne, United Kingdom; 1:10000) [47] [or polyclonal goat anti-DCX (sc-8066, Santa Cruz, Leiden, the Netherlands; 1:800) [47] diluted in 0.05M tris buffer saline (TBS), pH7.6. The next morning, the sections were washed with 0.05M TBS and incubated for 2 hours at room temperature with the secondary antibodies (biotinylated sheep anti-mouse 1:200 (GE Healthcare, United Kingdom), goat anti-rabbit 1:200 (Vector Laboratories, CA, USA) or biotinylated donkey anti-goat 1:500 (kindly provided by Dr. I. Huitinga, Netherland Institute for Neuroscience, Amsterdam)) diluted in 0.05M TBS, pH 7.6. Next, all sections were washed with 0.05M TBS and incubated for 90 minutes with avidin-biotin (ABC kit, Elite Vectastain, Brunschwig Chemie, Amsterdam, the Netherlands) diluted 1:800 in 0.05M TBS, in 0.3% triton at room temperature. Sections were then rinsed in 0.05M Tris-buffer (TB), pH7.4 and incubated for 6 minutes in 3–3’ diaminobenzidine (DAB, 20 mg/100 ml of Tris buffer, 0.01% H2O2). Finally, the sections were dehydrated, incubated in xylene for 10 minutes and dried for 2 days at room temperature.

#### Quantification

We quantified the number of Ki67-, BrdU- and DCX-positive cells that were located bilaterally along the rostral-caudal hippocampal axis in every 6th coronal section. In order to get an estimation of the total number of all positive stained cells along the entire rostral-caudal axis of the dentate gyrus (DG), the total number of the counted cells was multiplied by six, to correct for the fact that we only used one of six consecutive sections [44]. BrdU and Ki67-positive cells were quantified manually using a light microscope (40 x objective, Zeiss). Due to the complexity and number of DCX-positive cells, these cells were quantified by systematic random sampling using a Stereo investigator system (Microbrightfield, Magdeburg, Germany). Contours were drawn for each hippocampal section and the cells were counted using an optical fractionator at 40x magnification (Axiophot, Zeiss-West Germany). Optical fractionator settings were the same for all cells as follows: 70 x 80 counting grid size and 25 x 25 counting frame size. The same volume was sampled for every subject.

Cells were counted along the transversal axis, namely the suprapyramidal blade and the infrapyramidal blade and we further made a distinction between the rostral and caudal part of the dentate gyrus based on the curvature of the hippocampus. Although literature does not support evidence for an accurate distinction between the rostral and the caudal part of the DG, sections with bregma -1,34 to -2,70 were defined as the rostral dentate gyrus while the sections between bregma point -2,80 to -3,80 were defined as the caudal part of the dentate gyrus [48]. To correct for the variation in the amount of sections between the animals, in all cases seven sections were selected per animal.

### Statistical analysis

Analyses were performed using statistical package SPSS 17.0 for Windows XP. Data were normally distributed as determined by Shapiro-Wilk tests for normality and we therefore performed parametrical statistical analyses on datasets. Only for corticosterone levels we used a non-parametric statistical analysis (Kruskal Wallis followed by a Mann Whitney U test), as this dataset was not normally distributed. For datasets in which no time-effect was present we performed a two-way ANOVA (with CUS as the predicting factor and genotype as the moderating factor). If an interaction effect was significant a post hoc Fishers Least Significant Difference (LSD) test was performed, after determining equality of variances using Levene’s test (which was the case unless stated otherwise). As we always performed four post-hoc comparisons (control / non-stressed vs control / stressed or vs MR / non-stressed; control / stressed vs. MR / stressed; MR / non-stressed vs MR / stressed) we adjusted the alpha and considered p ≤ 0.0125 to be significant. Data are presented as mean with standard error of the mean (SEM). To compare the distribution of dendrites, we performed repeated-measures ANOVA on Sholl plots, with “distance from the soma” as within-subjects factor and “genotype” and “treatment” as fixed between-subject factors. A Pearson correlation was performed to analyze the relation between the number of BrdU- and Ki67-positive cells, and the relation between body weight and adrenal weight.

## Results

### Animals

Data for this study were obtained from 139 male mice, of which 73 were MR-tg and 66 were (littermate) controls. These mice belonged to 60 mixed gender litters with on average 7 pups.

### Neuroendocrine parameters

**Fig 1:**
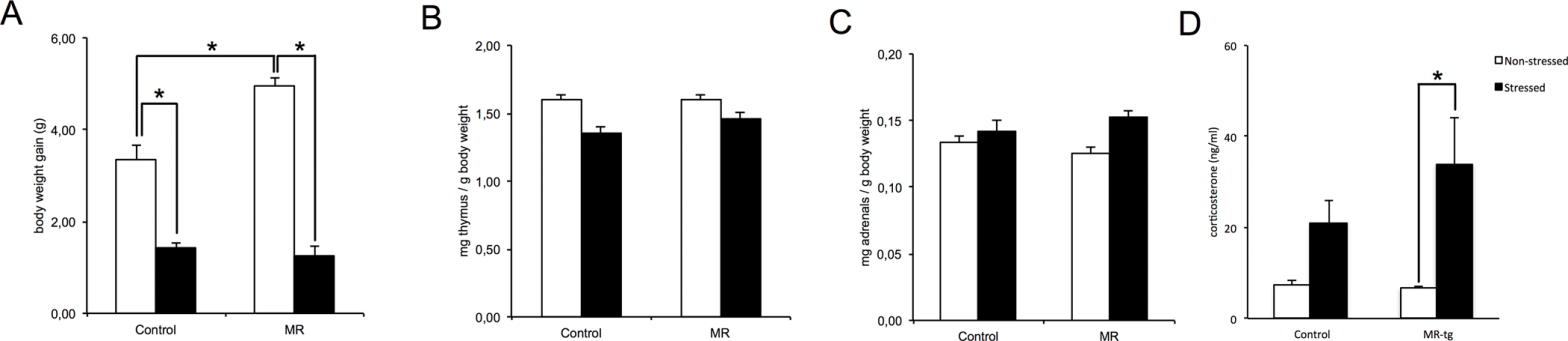
Neuroendocrinological parameters to determine the effectiveness of the CUS paradigm. These parameters included: (A) body weight gain (g), (B) thymus weight (mg / g body weight), (C) adrenals weight (mg / g body weight and (D) corticosterone levels. Data are expressed as mean ± SEM with p-values based on post-hoc LSD. n≥20 mice per group for A,B and C and n = 6–8 for D. *: significant, p ≤ 0.0125.

For bodyweight gain over 21 days of chronic unpredictable stress (CUS), there was a significant interaction between CUS and genotype (F(1,127) = 16.093, p<0.002; Fig 1A). Follow-up analysis showed that stressed control (p<0.001) as well as MR-tg mice (p<0.001) had reduced body weight gains when compared to corresponding non-stressed control groups (Fig 1A). The apparent interaction was explained by the fact that non-stressed MR-tg mice were heavier than non-stressed controls (p< 0.001).

There was no interaction nor a main effect of genotype on thymus weight normalized to bodyweight. CUS had a main effect on normalized thymus weight (F (1,95) = 20.828, p<0.001; Fig 1B). For the adrenal weight, no interaction nor a main effect of CUS or genotype was found. There was no correlation between body weight and adrenal weight in the control non-stressed mice (n = 8, r = 0.352, p = 0.393).

We observed a statistically significant difference in corticosterone levels between the different groups (χ2(2) = 12.438, p = 0.006) with a mean rank score of 8.14 for MR-tg non-stressed, 9.43 for control non-stressed, 19.17 for MR-tg stressed and 17.40 for control stressed. Post-hoc analysis revealed that MR-tg stressed mice had increased basal corticosterone levels compared to MR-tg non-stressed mice (U(13) = 4.00, Z = -2.64, p<0.008, Fig 1D).

### Behavior

#### Contextual fear conditioning

In total 24 control (CUS: n = 12; non-stressed: n = 12) and 24 MR-tg (CUS: n = 12; non-stressed: n = 12) male mice were subjected to contextual fear conditioning, a highly stressful learning task which (among other regions of the brain) involves the hippocampus [49].

**Fig 2:**
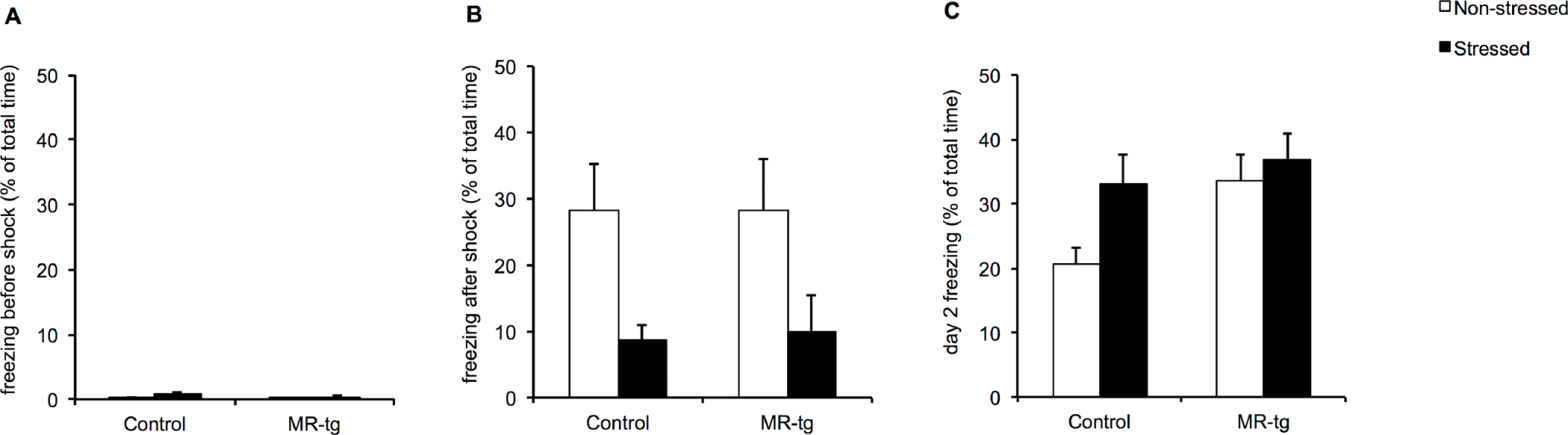
Contextual fear conditioning in stressed and non-stressed control and MR-tg mice. Bar graphs represent the results for: (A) freezing time before the foot shock on day 1, (B) freezing time after the foot shock on day 1, and (C) freezing time during the retention test on day 2. Data are expressed as mean + SEM with p-values based on post-hoc LSD. n = 12 mice per group.

Prior to the foot-shock, there was neither an interaction between stress and genotype nor a main effect of genotype. Chronic stress did affect freezing behavior prior to the foot-shock (F(1,45) = 6.720, p<0.013, Fig 2A), i.e. it increased freezing in this novel environment. However, it should be realized that the actual amount of freezing at this time of the test was very low, <1% of the total observation time.

Immediately after exposure to the foot-shock, mice displayed considerable freezing behavior (Fig 2B). CUS decreased freezing compared to controls immediately after receiving the foot-shock (F(1,45) = 9.483, p<0.04).

Twenty four hours later, when retention of the shock-context was tested, no interaction between CUS and genotype was discerned (Fig 2C). We did see a significant effect of CUS (F(1,45) = 0.165, p = 0.048). We also observed a significant effect of genotype (F (1,45) = 4.710, p = 0.036).

#### Object in-context recognition memory

**Fig 3:**
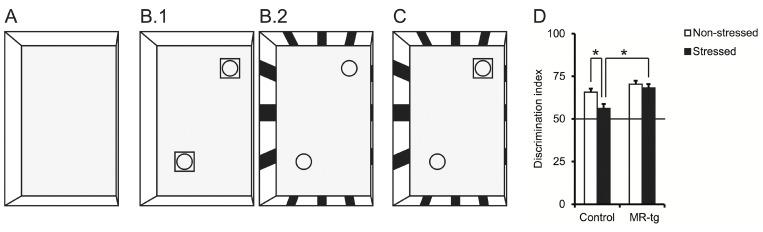
Object in context. Left: schematic representation to indicate the setup of the object-in-context experiment. A) Mice were initially habituated in a context that had no object. B1) During training, mice were placed in the same context but with two identical objects and then placed in a novel context with two identical novel objects (B2). (C) Finally the mice were placed in the latter context but with one object being replaced by an object from the first context. (D) The discrimination index as observed from the object in context experiment. Data are expressed as mean ± SEM with p-values based on post-hoc LSD. n = 9 mice per group. *: significant, p ≤ 0.0125.

To probe hippocampal function under non-arousing circumstances [29] we tested mice (all groups n = 9) in an object-in-location memory task (Fig 3). On the testing day (day three), the DI was tested against chance level (50%) and it was found to be highly significant above 50% for both the control non-stressed mice (one-sample t-test, p<0.001) and the MR-tg non-stressed mice (one-sample t-test, p<0.001). A DI significantly above the chance level was also found in control stressed mice (One-sample T-test, p<0.047) and MR-tg stressed mice (one-sample t-test, p<0.001). We observed an interaction between CUS and genotype at trend-level F(1,36) = 2.568, p = 0.1, Fig 3D). Follow-up post-hoc analysis revealed a significant memory deficit after CUS (compared to non-stress) in control mice (p<0.012), but not in MR-tg mice. Furthermore, MR-tg stressed mice perform significantly better than control stressed mice (p<0.003).

### Structural plasticity

We next examined CUS-induced changes in structural plasticity in specific hippocampal subareas that were previously reported to be very sensitive to chronic stress; that is, the apical dendritic complexity of CA3 pyramidal cells and adult neurogenesis in the dentate gyrus [37, 45].

#### Pyramidal cell morphology

For the parameters investigated, i.e. dendritic complexity, dendritic length and the number of branch points in CA3 apical dendrites, no significant interaction effects or effects of genotype were observed. We did find a significant main effect of CUS on the dendritic complexity index (F(1,30) = 5.319, p<0.029) and dendritic length (F(1,30) = 4.465, p<0.044), though not on the number of dendritic branch points (S2 Fig), which is largely compatible with what has been reported previously in other species [37, 50].

**Fig 4:**
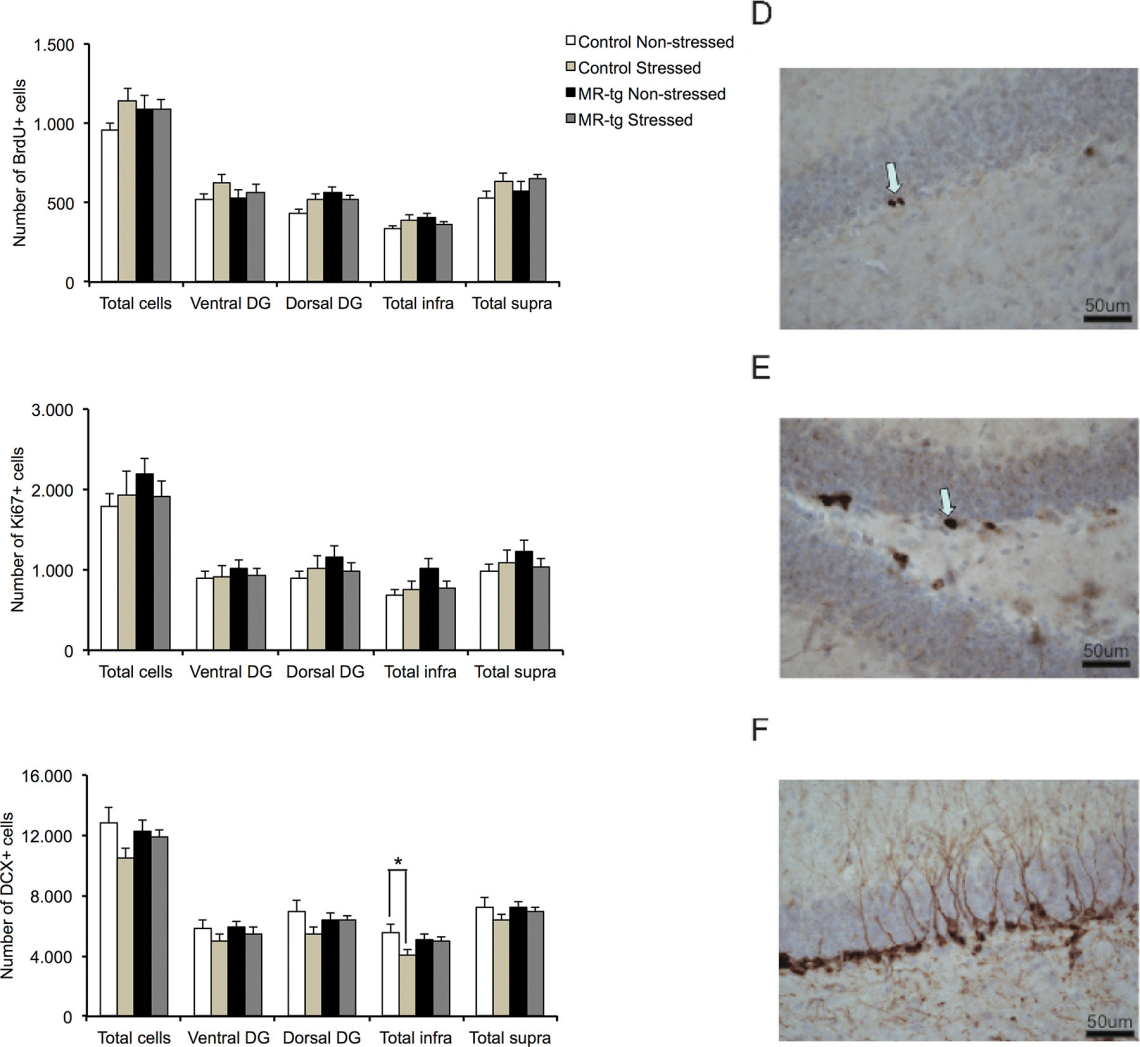
Sholl plots in stressed and non-stressed control and MR-tg mice. (A) The average dendritic length measured at specific distance points from the soma in all experimental groups. Analysis was based on the same cell groups as used in S2 Fig. Data are expressed as mean ± SEM. (B) Representative image of a CA3 pyramidal neuron analyzed at various bins representing the distance from the soma. We restricted the analysis to the apical dendrites only. Calibration bar: 20 μm.

Analysis of the average dendritic length measured at distinct distance points from the soma (Fig 4A) showed an overall significant difference over the several points from the soma (repeated measures Anova, sphericity assumed correction: F(15,390) = 301.782, p<0.001) but no significant dendritic length point x treatment x genotype effect was found (repeated measures Anova, sphericity assumed correction: F(15,390) = 1.294, p<0.266).

#### Neurogenesis

**Fig 5:**
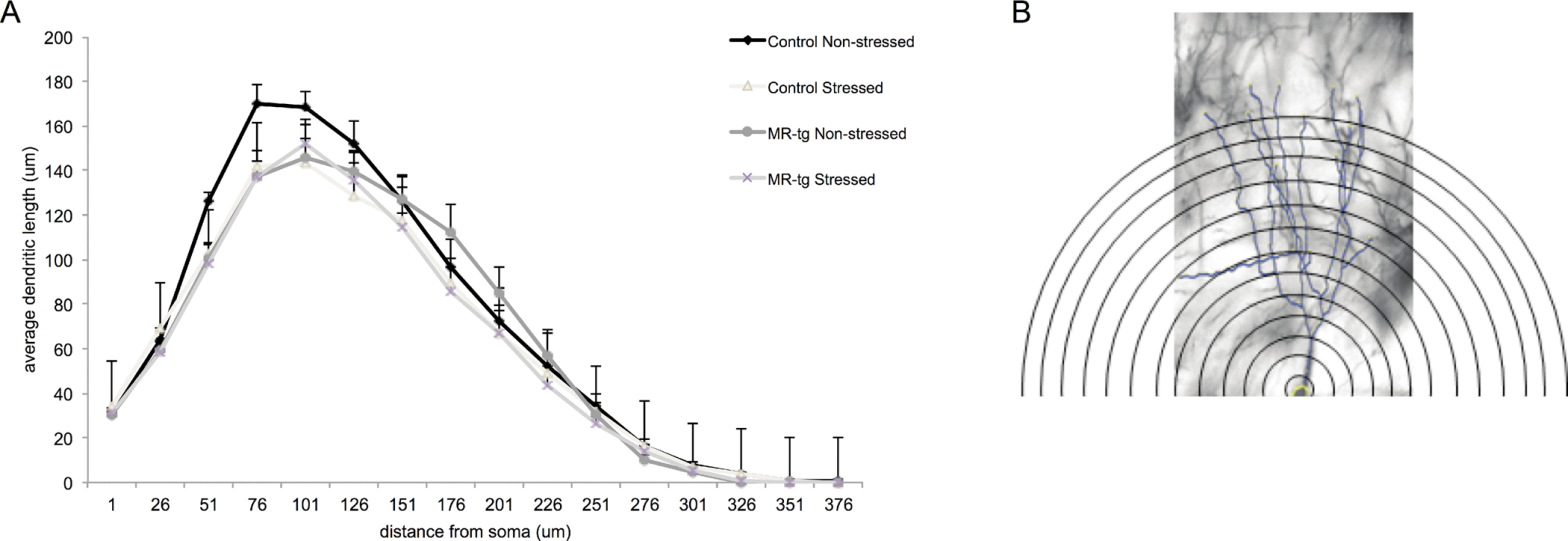
Neurogenesis results in stressed and non-stressed control and MR-Tg mice. Right panel shows representative examples of BrdU, Ki67 and DCX labeled cells being scored (D-F, arrows, calibration bar: 50 μm). Left panel shows the bar graphs on the left summarize the averaged results for: (A) BrdU positive cells, (B) Ki67 positive cells and (C) DCX positive cells. For each marker we analyzed the total number of cells, the number of cells along the rostral and caudal subareas of the hippocampus as well as the numbers for two subregions that make up this total number (total infra and total supra). Data are expressed as mean ± SEM with p-values based on post-hoc LSD. n = 8 animals per group. *: significant, p ≤ 0.0125.

BrdU-labelling was studied after a 3 week survival period (Fig 5A). Hence, the current BrdU numbers most likely reflect a combination of cell proliferation and survival [41]. Interestingly, we found a correlation that was highly significant (R2 = 0.516, p<0.005) between the total number of cells positive for BrdU and for Ki67 (Fig 5B), a marker for cell proliferation at the time of analysis. DCX labels immature neurons (Fig 5C), which is commonly used as a marker for adult neurogenesis.

When taking all immunostainings into account, we only found an interaction effect at trend level for BrdU, but not for Ki67 or DCX. For none of the markers did we observe an effect of genotype. A main effect of CUS was apparent for the total number of DCX-positive cells (F(1,31) = 4.274, p<0.048).

It has been argued that neurogenesis is not homogeneous across the DG. Along the transversal axis, for instance, neurogenesis in the suprapyramidal blade showed greater experience-related activity than the infrapyramidal blade [51]. We therefore refined our analysis and made a distinction for the three markers between the infra- and suprapyramidal blades. For BrdU, we observed a main effect of CUS in the suprapyramidal blade (F(1,31) = 5.268, p = 0.030). No effects were found in terms of Ki67. Effects in DCX staining were more pronounced: a significant interaction effect of CUS and genotype was present in the infrapyramidal blade (F(1,31) = 4.210, p = 0.05). Post-hoc analysis indicated that CUS caused a significant decrease in the number of DCX-positive cells in control mice (p<0.012) but not in MR-tg mice.

The rostral and caudal part of the hippocampus have been shown to be associated with distinct behaviors [52]. Therefore, we assessed neurogenesis along the rostral-caudal part of the DG for all markers (Fig 5). In the caudal subarea, our results revealed a significant genotype effect (F(1,31) = 4.413, p<0.045) for the BrdU-positive cells. No significant effects were found for the BrdU-positive cells in the rostral subarea. Main or interaction effects in Ki67-positive cells and DCX-positive cells along the rostral-caudal axis of the DG were not detected.

## Discussion

Environmental factors such as repetitive exposure to stressful events are an important risk factor for stress-related psychopathology; this can be moderated by genetic susceptibility or resilience factors [5]. It has been suggested that efficient MR function may confer resilience to stress-related psychopathology and cognitive decline [16–17]. Here we used a mouse model to specifically examine potential moderating effects of MR overexpression on CUS-induced behavioral deficits and structural plasticity.

We found that MR overexpression may indeed protect against CUS-induced deficits in a relatively non-arousing memory task, but not in highly stressful learning conditions. Although we found that CUS induced CA3 pyramidal dendritic atrophy in the dendritic complexity index and dendritic length, these changes appeared to be unaffected by MR overexpression. CUS–as reported before- suppressed neurogenesis, which was moderated by MR in the infrapyramidal blade. We therefore tentatively conclude that MR overexpression may protect against some but not all CUS-induced changes in hippocampal behavior and structural plasticity.

### Experimental model

We used transgenic mice with forebrain specific overexpression of human MR under the control of a CaMKIIα promoter [14]. More specifically, these animals have a 3–4 fold MR mRNA increase in the hippocampus and 8-fold increase in amygdala. Immunohistochemical staining of MR showed more MR-positive neurons and greater intensity of staining in CA-1, CA-2, CA-3 of the dorsal blade of the dentate gyrus (DG), lateral septum and cortex compared to control mice; we confirmed this in the present set of animals (unpublished observation). MR-Tg was not detected in cerebellum, hypothalamus or peripheral tissues such as liver, lung and muscle [14].

The presently reported effect of MR overexpression on corticosterone levels is not in line with results from Mitra et al. [53], who reported that selective MR overexpression in the amygdala reduces stress-induced corticosterone secretion. This could be explained by the different models used to induce MR overexpression; that is, Mitra et al. [53] used viruses to overexpress the MR locally (only in the BLA) in adult rats. In this model, the HSV vectors that was to drive overexpression infect only approximately 30% of the neurons. Therefore, other areas contributing to anxiety-like behaviour and HPA axis activity, such as the hippocampus [54], were not affected, as opposed to the model we used. Moreover, viral MR overexpression occurred only during adulthood [53]. In our model, MR overexpression started around PND15 and remained present up until measurements in adulthood, which may have resulted in compensatory mechanisms.

Similar to what was reported before for fully-grown MR-tg mice [14], we found MR-tg mice to be significantly heavier than control mice, even at a relatively young age (~2 months old). Since MRs were overexpressed in the forebrain but not in hypothalamus (unpublished observations), this might indicate that forebrain MR play a substantial role in appetite and/or metabolic processes. Although this is of interest, it most likely did not affect our present findings, as we did not use food-rewarded tasks.

Both control and MR-tg mice exposed to CUS gained less bodyweight over the 21-days period when compared to non-stressed mice. In addition, CUS caused a reduced normalized thymus weight, increased basal corticosterone levels but had no effect on the adrenal weight. This shows the effectiveness of the 21-days stress paradigm, which was based on earlier described CUS protocols in rats [25–27, 44, 55].

### Behavioral results

Our behavioral observations are in line with earlier studies showing that CUS hampers contextual memory formation under non-stressful conditions but enhances contextual fear memory formation [12, 56–58]. Our data indicate that MR overexpression does not moderate the effects of CUS on contextual learning under high arousing (fear) conditions, as opposed to contextual learning under low arousing conditions. The most likely explanation is that high-arousing contextual fear conditioning is associated with high corticosterone levels, in a range generally involving GR activation [13, 59]. Corticosterone levels during low-arousing contextual learning are probably too low to largely activate GR and may depend more prominently on MR, and its involvement in behavioral reactivity towards novel spatial situations [60]. A second and less likely explanations is that mice had reached a ceiling in contextual fear memory. Interestingly, we observed a main effect of genotype in contextual fear conditioning which was particularly evident directly after re-exposure. This might be due to MR-dependent differences in overall anxiety. However, we did not observe genotype effects in the elevated plus maze, determined one day before contextual fear learning (S1 Fig). Alternatively, MR expression may influence the generalization of fear. This too is unlikely because MR-tg and control animals showed low levels of freezing when tested in a different context (data not shown). The tendency towards more pronounced freezing in non-stressed MR-tg (vs control) mice agrees with earlier studies in naïve mice reporting that pharmacological blockade of MR hampers retention of fear context [61] and hampers selective attention, executive function and working memory in men [20–22, 62–63].

With respect to a very sensitive hippocampus-dependent memory task under relatively non-arousing conditions [29] we found support for our hypothesis: overexpression of MR appears to protect against CUS-induced deficits. Of note, although the difference between chronically stressed and control animals was highly significant in wild type mice and absent in MR-tg animals, the interaction effect was only evident at trend level, so interpretation of the data should be done with care. The employed memory task, as other contextual tasks [64], might improve with a rise in circulating CORT levels; we cannot entirely exclude that putatively higher circulating CORT levels in MR-tg mice with a history of CUS contributed to the observed resilience. In contrast to the contextual fear conditioning paradigm, we found no support that MR overexpression by itself (under non-stressful conditions) improves memory for an object-in-location.

### Structural plasticity

The potentially protective effect of MR overexpression under non-arousing conditions could be explained by accompanying changes in structural or functional plasticity. We addressed the first possibility, by examining dendritic complexity of CA3 pyramidal cells and adult neurogenesis in the dentate gyrus, two parameters that are exquisitely sensitive to CUS [37, 44]. Chronic unpredictable stress has been reported to result in reduced hippocampal CA3 dendritic complexity [37, 50, 65–66]. Although the exact mechanism is unknown, elevated plasma corticosterone levels and altered glutamatergic tone have been implicated [67–69]. In agreement, we found a main effect of CUS on the dendritic complexity index and dendritic length. However, there was no indication for a moderating effect of MR overexpression. Therefore, there is no reason to assume that the improved object-in-location memory in MR overexpressing (compared to wild type) CUS mice is caused by changes in CA3 dendritic complexity. CA3 neurons, however, are not the only critical elements for good performance in this particular learning task, which would explain the disparate findings. CA1 or dentate neurons may be equally important for optimal task performance, but—in terms of dendritic complexity- these are far less sensitive to CUS than CA3 neurons [37].

Chronic stress has also been reported to reduce hippocampal adult neurogenesis [38, 70]. CUS or MR overexpression did not consistently change BrdU or Ki67 staining in the DG; interaction effects were not linked to significant post-hoc differences between specific groups. However, we did find a significant moderating effect of MR overexpression on CUS-induced DCX suppression in the infrapyramidal blade. This effect might contribute to our behavioral observations; spatial learning depends on as well as affects neurogenesis. Neurons in the suprapyramidal blade show greater experience-related activity but mature later than those in the infrapyramidal blade [51]. Changes in the infrapyramidal blade may therefore disproportionally contribute to the behavioral changes. No changes were found along the rostral-caudal part of the hippocampus in all three neurogenic markers. The effects of CUS on neurogenesis are believed to be mediated—at least in part—by elevated plasma corticosterone levels [45, 71]. It seems unlikely, though, that the protective effect of MR overexpression is caused by a reduction of stress-induced corticosterone levels; our neuroendocrine observations do not support this.

## Conclusions

In conclusion, this study supports that MR overexpression may confer resilience to the effects of prolonged stress exposure on some but certainly not all aspects of hippocampal memory performance and structural plasticity. Comparable observations have been made for other mediators of the stress response: genetic deletion of CRH also preserves learning and memory and neuronal integrity which is otherwise disrupted after exposure to chronic stress [71]. Collectively, these studies indicate that reducing vulnerability factors (such as CRH) or enhancing resilience factors (such as MR) might maintain brain function at an optimal level in the face of chronic stress exposure.

## Supporting Information

S1 Fig Elevated plus maze results as observed in stressed control and MR-tg mice. The bar graphs show results for: (A) total number of arm entries, (B) percentage of open arm entries and (C) percentage of time spent in the open arms. We observed no interaction effect of genotype and time nor a main effect of genotype, for any of the parameters analyzed. Data are expressed as mean ± SEM. n = 24 mice per group.
(TIFF)

S2 Fig Dendritic complexity results as observed in Fig 4. Dendritic morphology in stressed and non-stressed control and MR-tg mice. An example of an analyzed neuron is depicted in A. The dendritic complexity results included: (B) dendritic complexity index, (C) dendritic length and (D) number of branch points. Data are expressed as mean ± SEM; n = 6–8 animals per group.
(TIFF)
