# Correction: Overexpression of Mineralocorticoid Receptors Partially Prevents Chronic Stress-Induced Reductions in Hippocampal Memory and Structural Plasticity

**DOI:** 10.1371/journal.pone.0145706

**Published:** 2015-12-18

**Authors:** Sofia Kanatsou, Brenna C. Fearey, Laura E. Kuil, Paul J. Lucassen, Anjanette P. Harris, Jonathan R. Seckl, Harm Krugers, Marian Joels

The captions for Figs 4 and 5 are swapped. Please see the corrected Figs 4 and 5 here.

**Fig 4:**
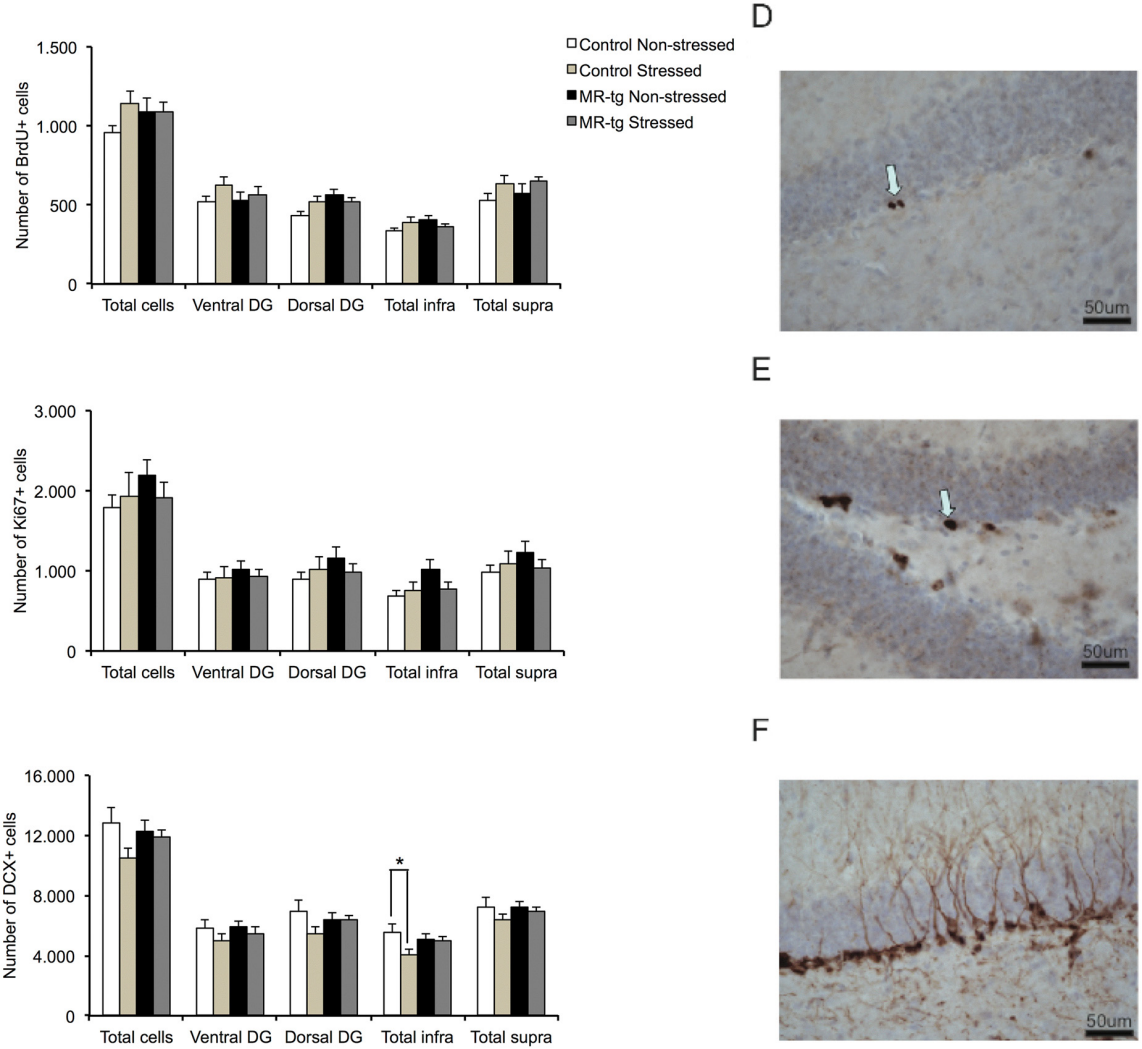
Neurogenesis results in stressed and non-stressed control and MR-Tg mice. Right panel shows representative examples of BrdU, Ki67 and DCX labeled cells being scored (D-F, arrows, calibration bar: 50 μm). Left panel shows the bar graphs on the left summarize the averaged results for: (A) BrdU positive cells, (B) Ki67 positive cells and (C) DCX positive cells. For each marker we analyzed the total number of cells, the number of cells along the rostral and caudal subareas of the hippocampus as well as the numbers for two subregions that make up this total number (total infra and total supra). Data are expressed as mean ± SEM with p-values based on post-hoc LSD. n = 8 animals per group. *: significant, p ≤ 0.0125.

**Fig 5:**
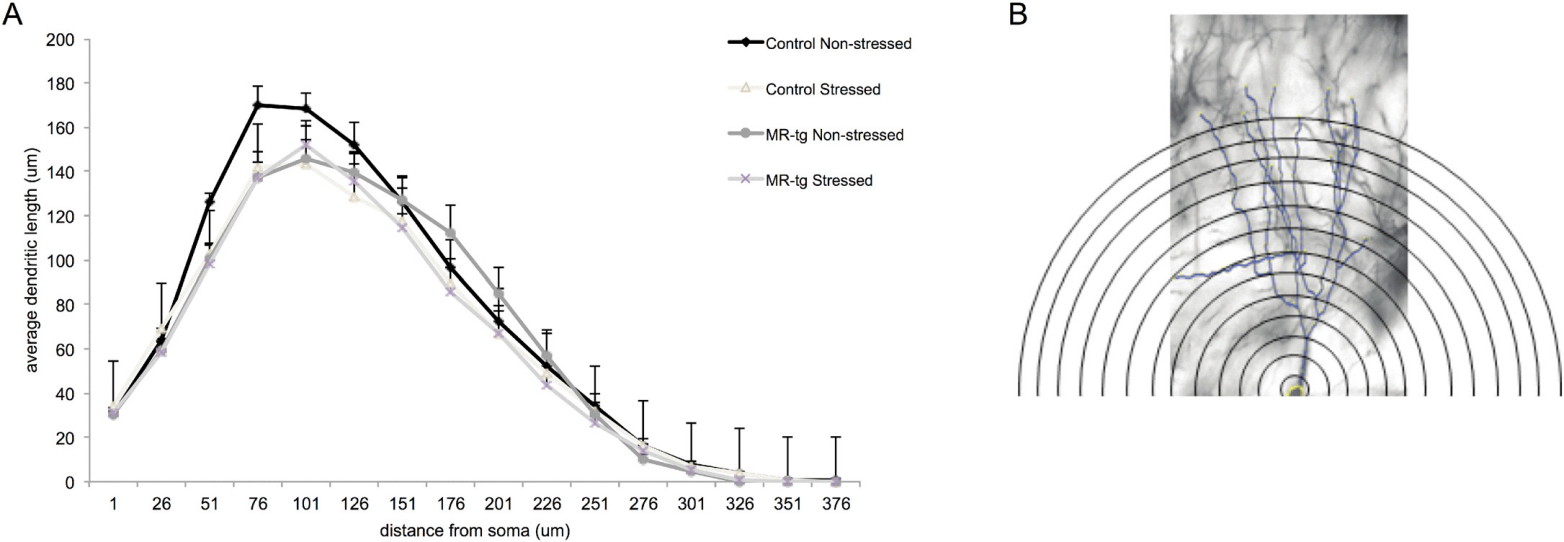
Sholl plots in stressed and non-stressed control and MR-tg mice. (A) The average dendritic length measured at specific distance points from the soma in all experimental groups. Analysis was based on the same cell groups as used in S2 Fig. Data are expressed as mean ± SEM. (B) Representative image of a CA3 pyramidal neuron analyzed at various bins representing the distance from the soma. We restricted the analysis to the apical dendrites only. Calibration bar: 20 μm.
