# Supplementary figures and images for: Overexpression of Mineralocorticoid Receptors Partially Prevents Chronic Stress-Induced Reductions in Hippocampal Memory and Structural Plasticity
Source: PLoS One. 2015 Nov 23;10(11):e0142012. doi: 10.1371/journal.pone.0142012 (PMC4658081; doi:10.1371/journal.pone.0142012)

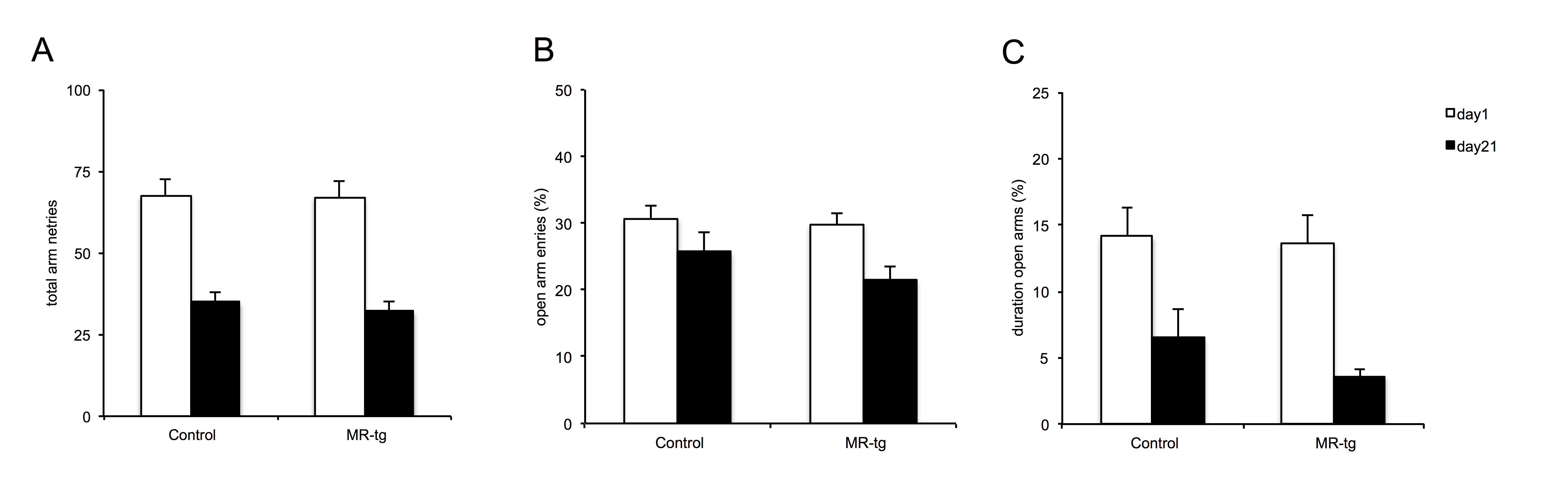

Supplement: S1 Fig — The bar graphs show results for: (A) total number of arm entries, (B) percentage of open arm entries and (C) percentage of time spent in the open arms. We observed no interaction effect of genotype and time nor a main effect of genotype, for any of the parameters analyzed. Data are expressed as mean ± SEM. n = 24 mice per group. (TIFF) [file pone.0142012.s001.tiff]
